# Effects of various artificial agarwood-induction techniques on the metabolome of *Aquilaria sinensis*

**DOI:** 10.1186/s12870-021-03378-8

**Published:** 2021-12-13

**Authors:** Ningnan Zhang, Shiyu Xue, Jie Song, Xiuren Zhou, Dahao Zhou, Xiaojin Liu, Zhou Hong, Daping Xu

**Affiliations:** 1 Research Institute of Tropical Forestry, Chinese Academy of Forestry, Guangzhou, 510520 China; 2 School of Life Science and Technology, Henan Institute of Science and Technology, Xinxiang, China; 3 Huazhou Yuanlai Agarwood Limited Company, Huazhou, 525100 China

**Keywords:** Brine drill treatment, Cold drill treatment, Fire drill treatment, Metabolites, Ethanol, Agarwood

## Abstract

**Background:**

Agarwood is a highly sought-after resinous wood for uses in medicine, incense, and perfume production. To overcome challenges associated with agarwood production in *Aquilaria sinensis*, several artificial agarwood-induction treatments have been developed. However, the effects of these techniques on the metabolome of the treated wood samples are unknown. Therefore, the present study was conducted to evaluate the effects of four treatments: fire drill treatment (F), fire drill + brine treatment (FS), cold drill treatment (D) and cold drill + brine treatment (DS)) on ethanol-extracted oil content and metabolome profiles of treated wood samples from *A. sinensis*.

**Results:**

The ethanol-extracted oil content obtained from the four treatments differed significantly (F < D < DS < FS). A total of 712 metabolites composed mostly of alkaloids, amino acids and derivatives, flavonoids, lipids, phenolic acids, organic acids, nucleotides and derivatives, and terpenoids were detected. In pairwise comparisons, 302, 155, 271 and 363 differentially accumulated metabolites (DAM) were detected in F_vs_FS, D_vs_DS, F_vs_D and FS_vs_DS, respectively. The DAMs were enriched in flavonoid/flavone and flavonol biosynthesis, sesquiterpenoid and triterpenoid biosynthesis. Generally, addition of brine to either fire or cold drill treatments reduced the abundance of most of the metabolites.

**Conclusion:**

The results from this study offer valuable insights into synthetically-induced agarwood production in *A. sinensis*.

**Supplementary Information:**

The online version contains supplementary material available at 10.1186/s12870-021-03378-8.

## Background

Agarwood is a fragrant resin-filled heartwood of the Aquilaria genus that is much more valuable on the global market than gold [1–3]. Agarwood has been used as a valuable incense in Buddhist, Hindu and Islamic practices in Asian countries. In China, it was employed as a traditional medicine in Ayurvedic practices [3, 4]. In addition, the primary active compounds of agarwood, which have been researched for neuroprotective, sedative, antibacterial and anti-inflammatory uses, have been observed in recent pharmacological and chemical studies [2, 5–7]. However, unless triggered by severe injuries or microbial infestation, a limited amount of agarwood can be produced by healthy Aquilaria trees. The production of agarwood is mainly done by wind or lightning injuries, or by insects and fungi nibbling [8, 9]. Asian traditional methods of agar wood production include cutting, nailing, boring holes, or cutting a piece of a trunk [3, 4, 9, 10]. This over-exploited wild *Aquilaria* plants, and several species are now dwindling and endangered [11, 12].

Traditional agarwood induction methods and non-traditional agarwood induction methods are the two most common types of agarwood induction methods available. Physical incursions such as burning, chisel drilling, and wounding with an axe are used in the traditional procedures, which have been in use in China for about 1000 years [13]. Other non-traditional induction strategies include trunk injection with solvents that contain ions or microbial additives to facilitate the production of agarwood [4, 11, 14] and trunk injection with solvents that contain ions but do not contain any microbial additives [4, 11, 14]. These induction methods are used in commercial agarwood production in China and other agarwood-producing countries. It follows that cultivating agarwood is vital to generating agarwood of excellent grade. Although some recent investigations imply that the quality of non-traditionally-produced agarwood is comparable to that of traditionally-produced agarwood, others have reported that there are chemical variations between the two types of agarwood [2, 4, 8, 11, 15].

Middle East and Southeast Asia [16] have a strong demand for agarwood as a raw resource, which is used for both incense and medicinal uses. The price of agarwood on the global market varies depending on its grade, spanning from $ 6000 per kilogram to $ 10,000 per kilogram [17]. Additionally, 1 kg of agarwood’s essential oil may be purchased for $ 30,000 [18]. The overall global market share of agarwood is estimated to be between $ 6 and $ 8 billion per annum. Traditional induction techniques are being investigated for the manufacture of agarwood, however such research is still in its early phases [4, 11]. Since there are different methods of agarwood production, a comparison of chemical constituents is absolutely required to gain comprehensive knowledge on quality and metabolite profiles of agarwood [8, 13]. Further studies are required to elucidate the mechanism of secondary metabolites accumulation in agarwood induced by non-traditional techniques. An increase in chromones is detectable on the 20th day after applying a treatment to *Aquilaria sinensis* [10, 15]. Majority of the chromones and the proportions of the composition are stabilized after 9 months of wounding. Although the process is unclear, these results indicate the complex dynamic nature of agarwood formation [8, 10, 15].

It was observed by Wang et al. [19] that salinity stress increases the production of 2-(2-phenylethyl) chromones and modulates the expression of new genes that are involved in signal transduction in the *Aquilaria sinensis* plant*.* The qualities of agarwood oils are influenced by the fragrance and longevity, resin content, geographic origin and purity [9, 20–22]. Essential oils typically arise from secondary metabolites with several hundreds of constituents. However, oil quantity and quality in agarwood are reported to be markedly dependent on agarwood induction techniques [8, 9, 21–23]. There have been prior studies on the metabolite profiles of agarwood [8]. Metabolites such as 2-(2-phenylethyl) chromones (PECs) are among the most important groups of aromatic compounds found in agarwood [5, 7, 24]. Chromone profiling using liquid chromatography-mass spectrometry (LC-MS) and nuclear magnetic resonance (NMR) separated chromones into three groups: tetrahydro-2-(2-phenylethyl) chromones (THPECs), epoxy-(2-phenylethyl) chromones (EPECs), and diepoxy-(2-phenylethyl) chromones (DEPEC). A large number of studies have indicated that both chromones and sesquiterpenes are essential for the medicinal and incense applications of agarwood [4, 8, 13]. Recently, it has been revealed that the quantity of chromones in agarwood fluctuates throughout agarwood production [25], but 2-(2-phenylethyl) chromones have been shown to accumulate significantly in high-quality agarwood [8, 13, 23, 25].

Several traditional methods may approximate the formation process of wild agarwood. However, traditional methods are laborious and time-consuming [4, 11, 23]. New non-traditional treatment methods have been reported to yield higher but slower accumulation of secondary metabolites and oil in agarwood [8, 15]. An extensive number of agarwood induction methods have been developed, including the cultured agarwood kit (CA-kit), the whole-tree agarwood inducing technique (Agar-Wit), and the biologically-induced agarwood induction technique (Agar-Bit). Using a combination of physical wounding and chemical induction, the CA-kit induces *Aquilaria* tree growth by injecting an inducing substance into the wound using an aeration device that is put into the wound [26, 27]. This method produces high-quality agarwood, but the impact of the procedures on human health and the environment are yet to be studied. Additionally, Agar-Wit creates a larger wound because the inducer is carried through plant vascular tissue by transpiration. The preloaded inducer in a transfusion set is spread through plant transpiration, resulting in a greater coverage area of agarwood but with more destroyed tissues [27, 28]. Specifically, Liu et al. [27] used a combination of methods, including a partially-pruned tree, a burning chisel-drilling tree, and fungi inoculation tree, to induce resin formation in *Aquilaria sinensis*. They discovered that the agarwood derived from the Agar-Wit induction method was of higher quality than agarwood derived from the existing methods. Similarly, the Agar-bit procedure distributes the inducing reagent through plant transpiration, with the exception that the reagents are injected directly into the tree’s stems [28], which results in a more uniform distribution of the reagent.

Previous research utilizing gas chromatography-mass spectrometry (GC-MS) or NMR have provided evidence for particular sesquiterpenes in agarwood [1, 29]. As a result, we conducted this study on the ethanol-extracted oil content of *A. sinensis* treated agarwoods and their metabolome profiles using various induction strategies. The findings provide vital information for future research into the commercial production of agarwood in *A. sinensis*.

## Methods

### Plant materials, treatment and sampling

Agarwood samples were produced from a 9-year-old *A. sinensis* plantation in Pingding Town, Huazhou City, Guangdong Province, China. No permissions are necessary to use such samples. The formal identification of the samples was conducted by Prof Daping Xu and no voucher specimens have been deposited. In total, 12 artificial agarwood induced by wounding based on fire drill treatment (F), fire drill + brine treatment (FS), cold drill treatment (D) and cold drill + brine treatment (DS) were investigated. Brine is a solution of sodium chloride having a mass fraction of 26%.

For the fire drill treatment: we heated the drill in the stove (the temperature was about 750 °C), and immediately started drilling the trunk. We used *A. sinensis* with a diameter of 10 cm at breast height and drilled through the trunk at a distance of 50 cm from the ground above the trunk. The hole diameter was 1.5 cm and the hole spacing was 25 cm (4 holes in total). For the fire drill + brine treatment, the wounded trunk was washed with brine after perforation.

For the cold drill treatment: a drill (no heated drill) was used to make a hole through the trunk at a distance of 50 cm from the ground. The hole diameter was 1.0 cm with 25 cm hole spacing (4 holes in total). For the cold drill + brine treatment, the wounded trunk was rinsed with brine after perforation. The woods were harvested 4 weeks after treatment for analysis [19].

**Fig. 1:**
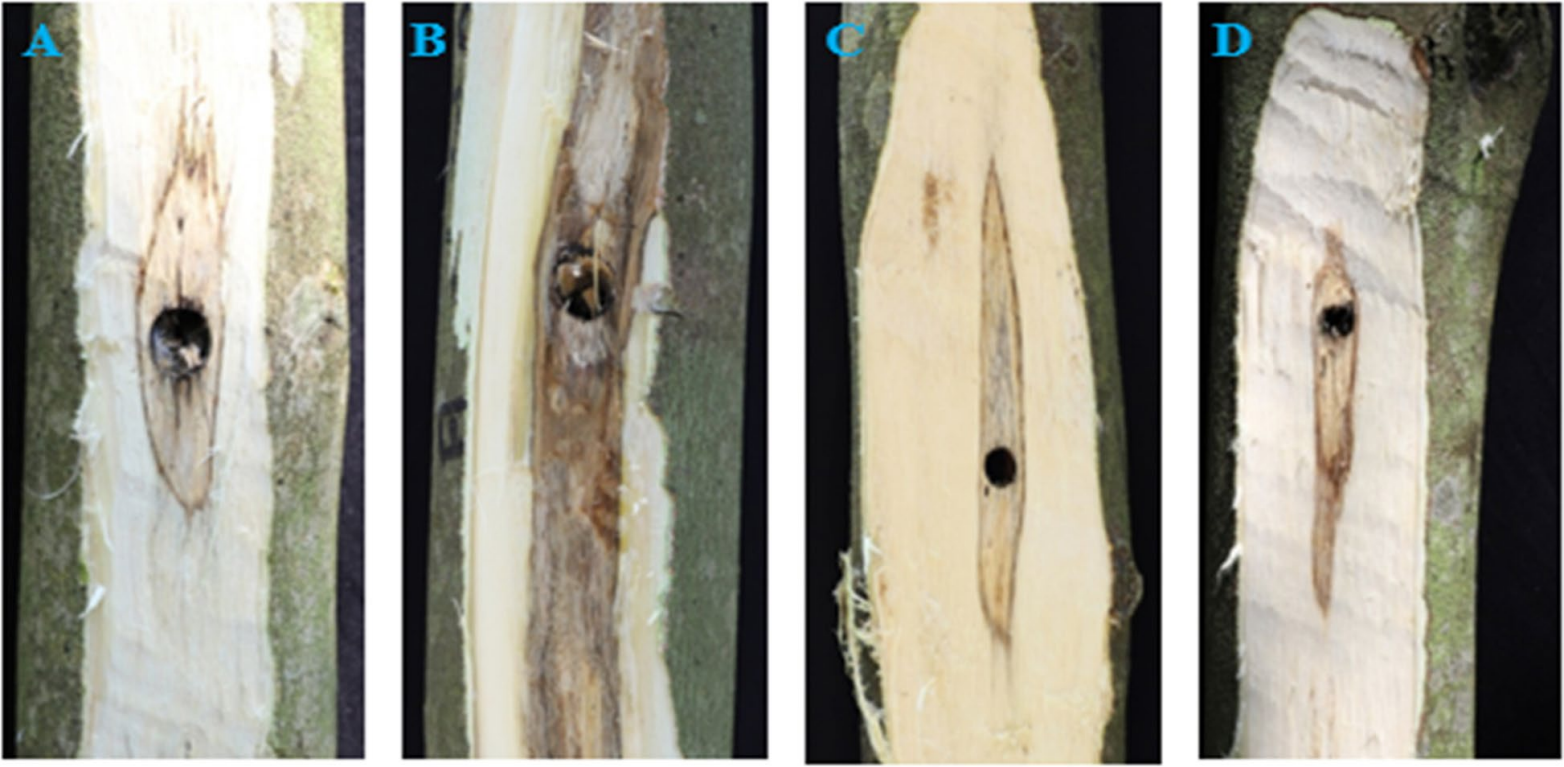
Wounds created in *A. sinensis* in this study. **A.** Fire drill treatment. **B.** Fire drill + brine treatment. **C.** Cold drill treatment. **D.** Cold drill + brine treatment

All treatments were applied to three different trees (biological replicates), involving 12 trees in this experiment. After treatments, the discolored parts of the trunks were sampled with a knife (Fig. 1). Samples (3 cm depth and radius 5 cm semi-circle) were taken at 5 cm below the punch sites. All wood samples were put into liquid nitrogen and transported to the laboratory. The wood samples were crushed into powder in a liquid nitrogen and placed in an ultra-low temperature refrigerator (− 80 °C) until the subsequent analyses were performed for use later.

### Ethanol extraction of oil from wood samples and quantification

Light and temperature exposure caused the wood samples to lose volatile compounds, therefore they were air-dried in a dark room and stored in a bag at a cool ambient temperature (4 **°**C). All botanical materials were size-reduced prior to the extraction procedure in order to optimize sample dispersion, heat transmission, access to the cells’ content, and the release of ethanol from the cells’ contents. The wood was cut into smaller pieces with a saw, and each piece of wood was shredded with a small knife once it had been cut. A Panasonic miller was used to crush the shredded wood in order to prevent the loss of volatile compounds in the final stage in the extraction process. Samples were extracted by an ultrasonic-assisted solvent method according to Samadi et al. [23]. Two grams (2 g) of samples were weighed, dried at 50 °C for 48 h to a constant weight, and water content was measured. The dried specimens were put into a 100 ml conical flask, and 50 ml of 95% ethanol was added. Under the conditions of 60 °C and 37 K Hz, the dried specimens were extracted for 30 min in an ultrasonic cleaner (Germany, ElmasonicP300H) twice. The mass loss was supplemented with 95% ethanol. After solution filtration, the 8 mL of filtrate (2 mL per tube) was evaporated in a Vacufuge plus Vacuum Concentrator (Germany, Eppendorf concentrator 5301). Then, the filtrate was evaporated at 60 °C for 3 h. After that, the dish was weighed, and the amount of ethanol-extracted oil in it was determined.

### Preparation of samples for metabolite extraction

Three grams (3 g) of the samples from the four treatments (three biological replicates (12 samples)) were lyophilized and then crushed into a powder using the MM400 mixer with zirconia beads with 15 mm for 1.5 min at 30 Hz. Samples of 100 mg powder were extracted in 1.0 mL overnight at 4 **°**C in 70% aqueous methanol and centrifuged for 10 min at 10000 rpm in order to absorb the extracts. The extracts were filtered and transferred into a fresh liquid chromatography-mass spectrometry (LC-MS) tube. The quality control (QC) analysis was conducted by combining sample extracts to verify changes in repeated analyses to validate the data. The analysis was done in accordance with earlier protocols [30].

### Metabolites detection and assessment

Comprehensive metabolites profiling with a wide variety of targets was carried out using the MetWare database (http://www.metware.cn) [31]. The metabolites were subjected to a qualitative analysis based on secondary spectral information. The triple quadruple-bar mass spectrometry in the multi-reaction monitoring (MRM) mode was also used to accomplish metabolite quantification. Principal components analysis (PCA) was used to visualize the variability between and within the groups in the data set. Orthogonal partial least squares-discriminant analysis (OPLS-DA) with a log2FC > 1 threshold and a variable importance of projection (VIP) allowed for the production of differentially accumulated metabolites (DAMs). Functional annotation of the DAMs was carried out [32] with the help of the KEGG database. An LC-ESI-MS/MS system was used to analyze the agarwood extracts for their metabolite profiles (HPLC, Shim-pack UFLC SHIMADZU CBM30A system, Kyoto, Japan; MS, Applied Biosystems 6500 Q TRAP, San Diego, CA, USA). The analytical conditions were adapted from Chen et al. [33]. The HPLC effluent was alternatively connected to an electrospray ionization (ESI)-triple quadrupole-linear ion trap–MS/MS system (Applied Biosystems 4500 Q TRAP, San Diego, CA, USA). Metabolite quantification was done using multiple-reaction monitoring (MRM) [34] and the MetWare MWDB database based on their standard metabolic operating procedures [35].

### Data analyses

First, quality control (QC) analysis was carried out to ensure that the data was reliable before proceeding with subsequent analyses. The quality control sample was made by combining agarwood samples to monitor the changes in repeated analyses. To conduct statistical analysis, agarwood samples were uploaded to AB Sciex’s Analyst program (version 1.6.1; AB Sciex, Canada), which contained data matrices with the total ion intensity of the metabolites. The orthogonal partial least squares discriminant analysis (OPLS-DA) was used for variable selection before submitting metabolites to KEGG analysis. When the variable importance in projection (VIP) parameter was used, the relative relevance of each metabolite to the OPLS-DA model was determined. VIP > 1 and fold change of 2 ≥ 0.5 were used as criteria [35]. The prcomp function in R (version 3.3.2; www.r-project.org) was used to create the PCA and Ward’s hierarchical clustering heatmaps [36]. A biosynthetic pathway was constructed in accordance with KEGG (https://www.genome.jp/kegg) [32]; and a pathway analysis was performed with the MetaboAnalyst (http://www.metaboanalyst.ca) based on the change in metabolite concentrations when compared to the controls [35].

## Results

### Influence of different treatments on ethanol-extracted oil content derived from Agarwood

**Fig. 2:**
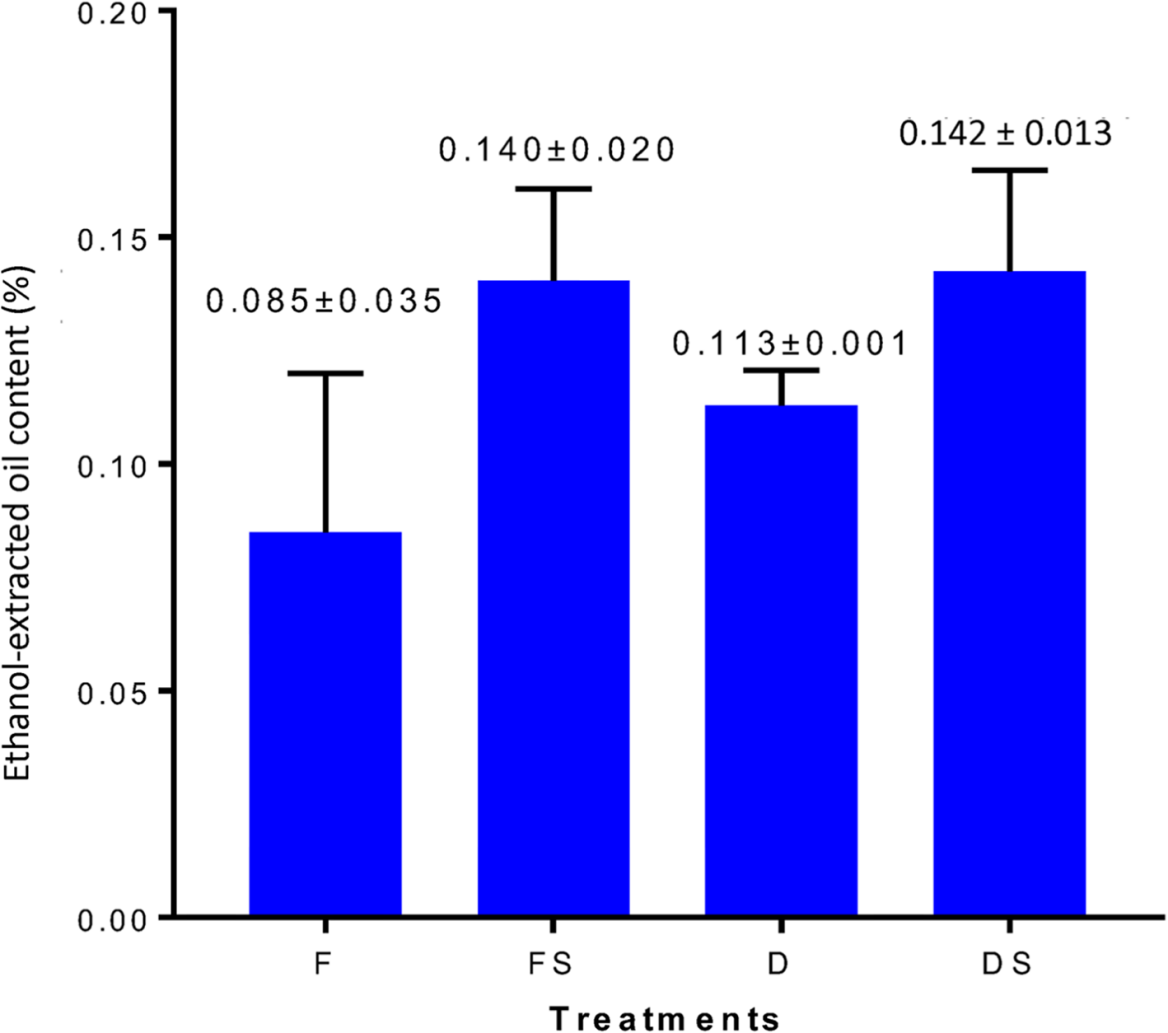
Variations in ethanol-extracted oil content in woods of *A. sinensis* subjected to four treatments (fire drill treatment (F), fire drill + brine treatment (FS), cold drill treatment (D) and cold drill + brine treatment (DS)). The bars represent the mean ethanol-extracted oil content across three biological repeats and error bars indicate standard deviation

We used the ultrasonic-assisted solvent extraction method with minor modifications [23] to extract and quantify ethanol-extracted oil content from the *A. sinensis* wounded by fire drill treatment (F), fire drill + brine treatment (FS), cold drill treatment (D) and cold drill + brine treatment (DS) (Fig. 1). The four treatments had on the ethanol-extracted oil content (%) in the order of DS (0.143 ± 0.022%) > FS (0.140 ± 0.020%) > D (0.113 ± 0.007%) > F(0.085 ± 0.035%) (Table S1; Fig. 2). This suggests that either addition of brine to either F or D increases the ethanol-extracted oil content in *A. sinensis* in relation to their counterparts without brine (F and D, respectively). Collectively, we infer that the different treatments affects the quantity and quality agarwood from treated *A. sinensis*.

### Targeted metabolome profiling of agarwood produced by four different induction techniques

The quantity and quality of agarwood produced from *A. sinensis* is highly influenced by stress imposed or microbial attack on the plant [37–39]. Since we observed an effect of the different treatments on the oil production in agarwood from *A. sinensis*, we further conducted a widely-targeted metabolome profiling on agarwood samples (4 treatments × 3 biological repeats) from *A. sinensis* using the Ultra-Performance Liquid Chromatography Tandem Mass Spectrometry (UPLC-MS/MS).

**Fig. 3:**
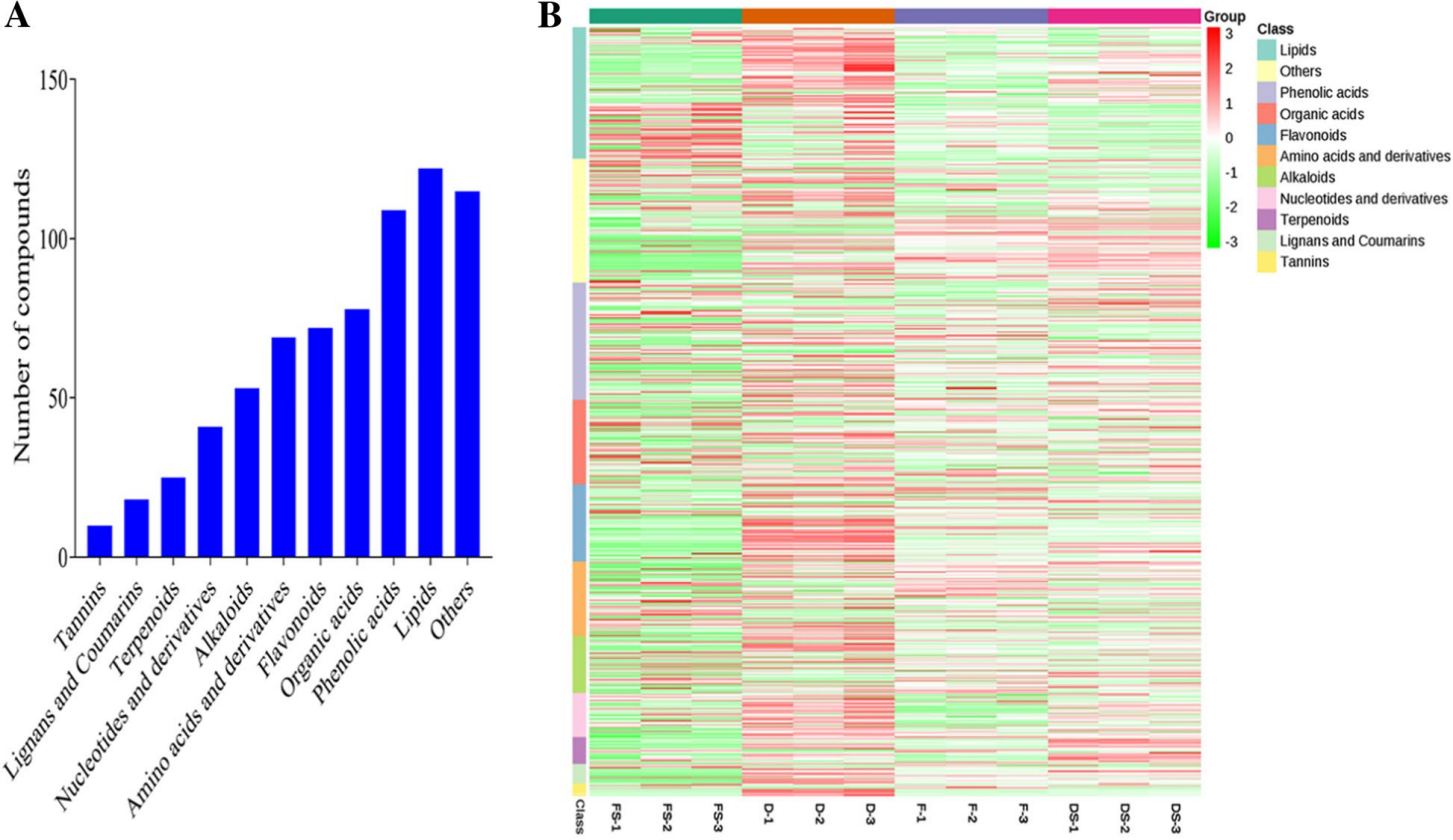
Metabolome profiling of the agarwood samples from *A. sinensis.* **A.** Classification of the 712 detected metabolites into 11 classes. **B.** Heatmap clustering showing correlation among the four treatments applied in this study. Green, chocolate, medium slate blue and magenta colors represent samples from fire drill treatment (F), fire drill + brine treatment (FS), cold drill treatment (D) and cold drill + brine treatment (DS), respectively. The class of compounds and their ion intensities are shown in the legend

From the UPLC-MS/MS, we detected a total of 712 compounds among the four treatments (F, FS, D and DS). These compounds consisted of 122 lipids, 109 phenolic acids, 78 organic acids, 72 flavonoids, 69 amino acids and derivatives, 53 alkaloids, 41 nucleotides and derivatives, 25 terpenoids, 18 lignans and coumarins, 10 tannins and 115 others (Fig. 3A). We subjected the ion intensities of the 712 metabolites into heatmap clustering and principal component analysis (PCA). The four treatments and their biological repeats were clustered into two main groups (Fig. 3B). The Cluster I comprised only samples from FS whereas the Cluster II comprised F, D and DS. This trend suggests that FS is relatively unique compared with the other three treatments. The results of PCA indicate that 79.89% variability in the 712 metabolites could be explained by the first 3 principal axes) among the 12 samples with each treatment and its samples grouped together (Fig. S1A-C). The grouping pattern of the biological replicates observed in heatmap clustering and PCA indicates the high quality of metabolome profile in this study.

### Identification of the differentially accumulated metabolites in the four-agarwood produced from *A. sinensis*

**Fig. 4:**
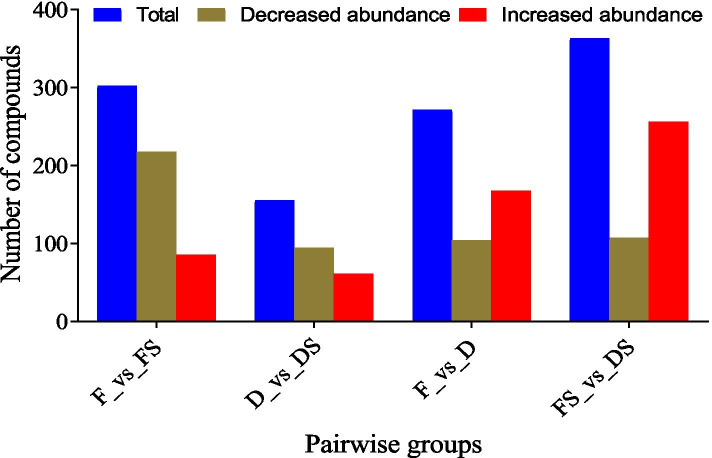
Differentially accumulated metabolites detected among the four pairwise groups of the agarwood from *A. sinensis.* The four samples obtained from fire drill treatment (F), fire drill + brine treatment (FS), cold drill treatment (D) and cold drill + brine treatment (DS)

**Fig. 5:**
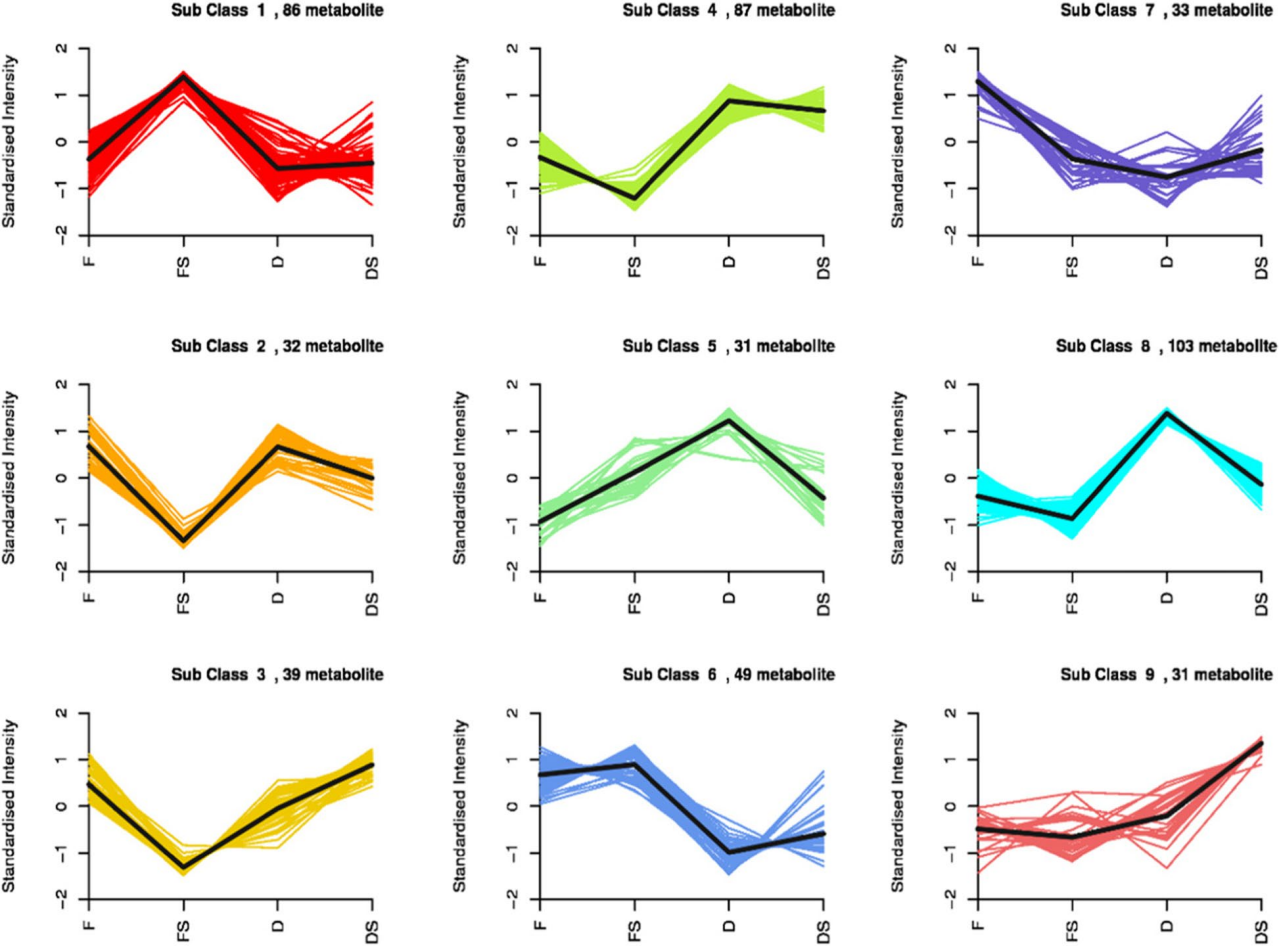
K-means clustering of differentially accumulated metabolites based on standardized ion intensities of metabolites (log10 transformed) from agarwood induced by fire drill treatment (F), fire drill + brine treatment (FS), cold drill treatment (D) and cold drill + brine treatment (DS) in *A. sinensis*. The sub class and metabolite numbers are displayed on top of each plot. The colored lines represent the standardized ion intensity while the black line represents the mean value of the standardized ion intensity of metabolites in each sub-class

We further subjected the 712 compounds to a differential accumulation analysis in pairwise comparisons using discriminant analysis of OPLS-DA at a threshold of log2FC ≥ 1 and variable importance in projection (VIP) ≥ 1. From this analysis, a total of 302, 155, 271 and 363 differentially accumulated metabolites (DAM) were obtained in F_vs_FS, D_vs_DS, F_vs_D and FS_vs_DS, respectively (Fig. 4; Supplementary Table S2 A-D). The majority of DAMs in F_vs_FS and D_vs_DS decreased in abundance (Fig. 4). This implies that the addition of a brine treatment decreased the extent of metabolites accumulation compared to their controls (F and D) (Table 1). This trend was largely consistent with results from the K-means clustering (Fig. 5). Conversely, most of the metabolites increased in abundance in D and DS samples compared to F and FS, respectively (Fig. 4; Table 1). This suggests that cold drill improves accumulation of metabolites compared to fire drill treatment.

**Table 1:** Number of compounds from the eleven classes and their ion intensity identified in the agarwood from *A. sinensis* induced by different treatments

Class	F		FS		D		DS	
	NoC ^a^	Ion intensity	NoC ^a^	Ion intensity	NoC ^a^	Ion intensity	NoC ^a^	Ion intensity
Alkaloids	36	52,797,606.1	33	14,537,574.3	21	32,346,979.27	32	49,146,242
Amino acids and derivatives	48	85,165,228.4	33	37,596,695.1	26	18,366,374.57	31	78,632,779
Flavonoids	58	69,920,663.7	20	3,072,415.9	45	39,081,955.6	34	5,319,031.6
Lignans and Coumarins	16	15,223,715.3	10	7,486,259.7	9	14,401,600	8	7,593,291.7
Lipids	54	28,322,568.1	34	43,464,023.1	28	20,040,812.83	8	784,199.87
Nucleotides and derivatives	27	9,091,952.97	13	5,787,767.2	15	17,270,308.37	1	112,305.33
Organic acids	35	35,139,215.6	20	6,451,408.83	24	55,120,567.43	9	9,179,028.9
Others	68	203,317,831	28	29,031,355.1	35	108,010,478.6	17	28,693,518
Phenolic acids	64	16,813,920.6	31	6,117,806.1	49	9,649,368.833	29	5,248,732.2
Tannins	9	388,543.367	3	86,313.2333	6	4,109,359.633	5	513,901.37
Terpenoids	12	17,555,711.7	3	1,092,603.33	12	41,771,620.83	5	65,389,197
Total	427	–	228	–	270	–	179	–

(A) Fire drill treatment (F). (B) Fire drill + brine treatment (FS). (C). Cold drill treatment (D) and (D). Cold drill + brine treatment (DS)

^a^Number of compounds in each class

We conducted a comparative analysis among the four pairwise groups and identified conserved DAMs (Fig. S2). This indicates that the four treatments have some levels of similarity in terms of induced metabolome changes in agarwood from *A. sinensis* (Fig. 3B).

### KEGG pathway enrichment analyses of differentially accumulated metabolites from agarwood

**Fig. 6:**
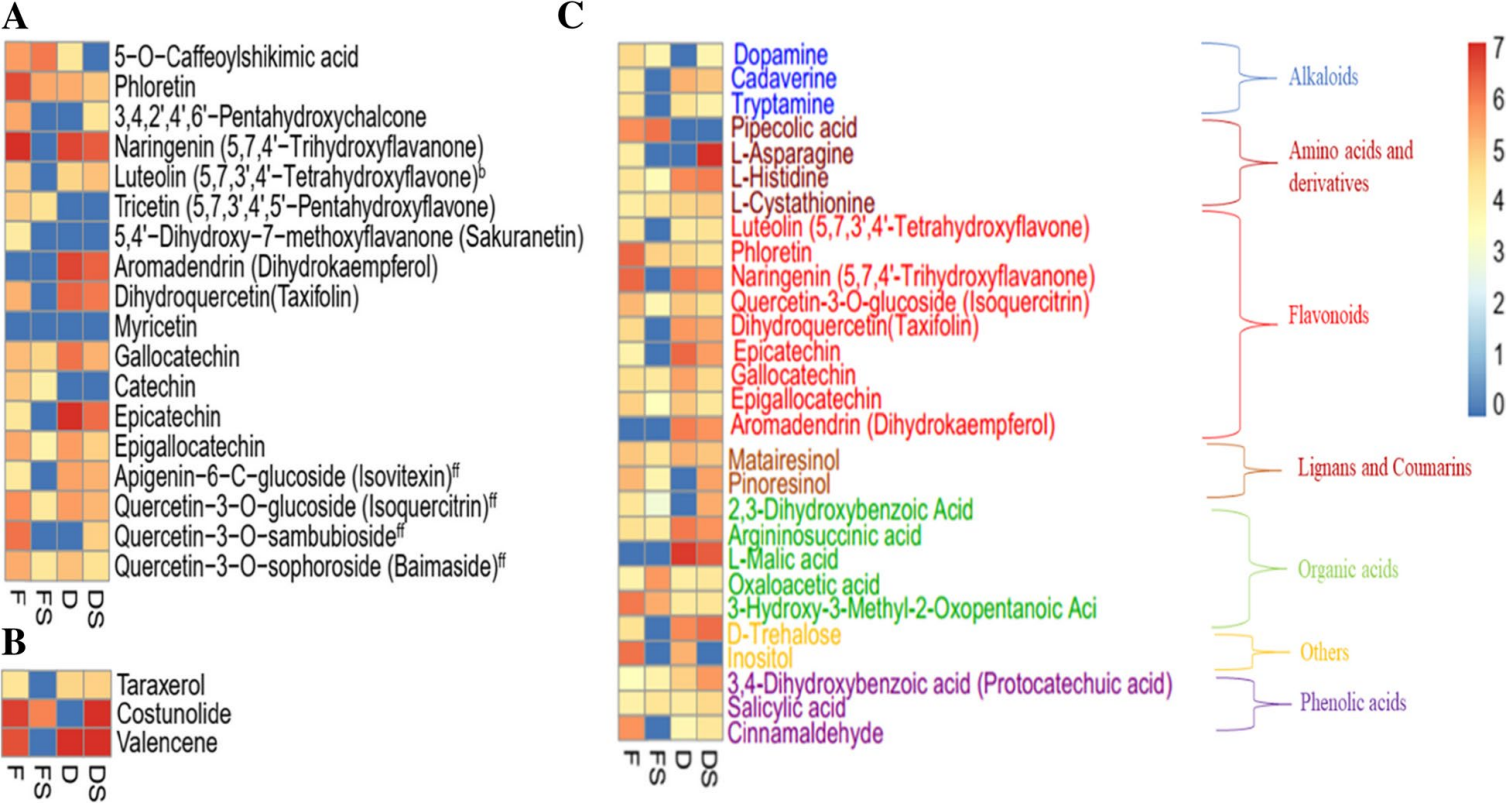
Differentially accumulated metabolites involved in enriched pathways detected among the four treatments (fire drill treatment (F), fire drill + brine treatment (FS), cold drill treatment (D) and cold drill + brine treatment (DS)) in agarwood from *A. sinensis*. **A** Flavonoid/flavone and flavonol biosynthesis. Compounds with superscripts *ff* are involved in flavone and flavonol biosynthesis whereas *b* is involved in flavonoid/flavone and flavonol biosynthesis. **B** Sesquiterpenoid and triterpenoid biosynthesis. **C** Biosynthesis of secondary metabolites. Metabolite abundance levels (ion intensities) in the four treatments (F, FS, D and DS) were log10 transformed. Those with red color indicates high abundance whereas deep to sea blue denotes absence or low accumulation of metabolites (see legend on right side of the figure)

We performed a KEGG pathway enrichment analysis with the DAMs detected in the pairwise groups at *p*-value < 0.05. Flavonoid biosynthesis was detected as a common pathway involved in the four pairwise groups with 14 unique metabolites (Table 1; Fig. 6A). Ten of these metabolites were highly accumulated in F samples; six flavonoids were highly accumulated in FS and nine flavonoids were highly accumulated in either D or DS (Fig. 6A). For instance, 5-O-Caffeoylshikimic acid was accumulated in FS > F > D (Fig. 6A). Another example is Naringenin which was accumulated relatively equally in F, D and DS but absent in FS (Fig. 6A). These results suggest the involvement of flavonoid biosynthesis pathway in regulating the quality of agarwood produced from *A. sinensis*.

In addition to flavonoid/flavone and flavonol biosynthesis elaborated above, three metabolites from the sesquiterpenoid and triterpenoid biosynthesis were differentially accumulated and induced by the treatments. For instance, Taraxerol, Costunolide and Valencene were in higher abundance in F and DS compared to either FS or D which lacked at least one of these compounds (Table 2; Fig. 6B), suggesting that only fire and cold drills with brine induced key compounds involved sesquiterpenoid and triterpenoid biosynthesis.

**Table 2:** Enriched KEGG Pathways among the pairwise groups

KEGG pathway	KO_ID^a^	F_vs_D		FS_vs_DS		F_vs_FS		D_vs_DS	
		DAMs^b^	*P*-value	DAMs^b^	*P*-value	DAMs^b^	*P*-value	DAMs^b^	*P*-value
Flavonoid biosynthesis	ko00941	12	< 0.001	–	–	8	0.043	8	< 0.001
Flavone and flavonol biosynthesis	ko00944	–	–	–	–	–	–	5	0.033
Sesquiterpenoid and triterpenoid biosynthesis	ko00909	–	–	–	–	3	0.042	–	–
Biosynthesis of secondary metabolites	ko01110	–	–	–	–	–	–	28	0.044

^a^Kyoto Encyclopedia of genes and genomes (KEGG)

^b^Number of differentially accumulated metabolites. Agarwood from *A. sinensis* obtained from fire drill treatment (F), fire drill + brine treatment (FS), cold drill treatment (D) and cold drill + brine treatment (DS)

A total of 28 compounds comprising two alkaloids, four amino acids and derivatives, nine flavonoids, two lignans and coumarins, five organic acids, two others and three phenolic acids were found in the enriched pathways of the biosynthesis of secondary metabolites (Table 2; Fig. 6C). Most of the metabolites decreased in abundance in FS compared to F. However, the opposite trend was observed in DS relative to D.

Collectively, the KEGG pathway enrichment analysis revealed that flavonoid/flavone and flavonol biosynthesis, sesquiterpenoid and triterpenoid biosynthesis could be targeted for pathway engineering to improve the quantity and quality of agarwood produced from *A. sinensis*. Besides, it also confirms the differential effects of the four treatments on the agarwood metabolome and quality.

### Accumulation of 2-(2-phenylethyl) chromones and derivates known to be involved in agarwood formation and quality attributes

**Fig. 7:**
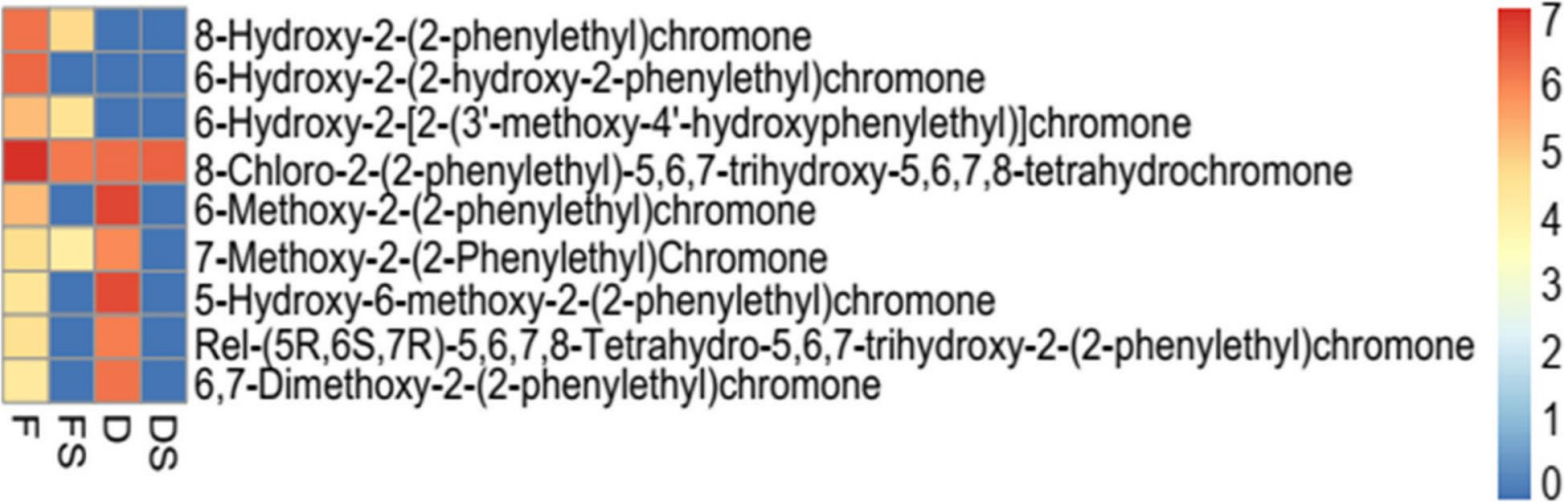
Heatmap of nine 2-(2-phenylethyl) chromones and derivates detected among differentially accumulated metabolites in the four treatments (fire drill treatment (F), fire drill + brine treatment (FS), cold drill treatment (D) and cold drill + brine treatment (DS)). Those with red color indicates high abundance whereas deep to sea blue denotes absence or low accumulation of metabolites (see legend on right side of the figure). The abundance level is the average of the three biological repeats and average levels were log10 transformed

A number of previous studies reported that 2-(2-phenylethyl) chromones and derivates are key components in agarwood formation and quality [29, 40–43]. For a practical genetic improvement, it has been shown that 2-(2-phenylethyl) chromones and derivates were the key biomarkers for agarwood formation in *A. sinensis* [8]. Therefore, we specifically screened for 2-(2-phenylethyl) chromones and derivates among the DAMs detected in F_vs_FS, D_vs_DS, F_vs_D and FS_vs_DS. We detected a total of nine 2-(2-phenylethyl) chromones and derivates (Fig. 7). All the nine compounds were present in the F with the most accumulated being 8-hydroxy-2-(2-phenylethyl) chromone, 6-hydroxy-2-(2-hydroxy-2-phenylethyl) chromone and 8-chloro-2-(2-phenylethyl)-5,6,7-trihydroxy-5,6,7,8-tetrahydrochromone. Unlike F, the FS treatment had only four compounds accumulated (8-Hydroxy-2-(2-phenylethyl) chromone, 6-hydroxy-2-[2-(3′-methoxy-4′-hydroxyphenylethyl)] chromone, 8-Chloro-2-(2-phenylethyl)-5,6,7-trihydroxy-5,6,7,8-tetrahydrochromone and 7-methoxy-2-(2-Phenylethyl) chromone. Hence, we speculate that F treatment induces a higher agarwood formation with better quality attributes than FS. Conversely, six compounds were identified in D with exception of 8-Hydroxy-2-(2-phenylethyl) chromone, 6-Hydroxy-2-(2-hydroxy-2-phenylethyl) chromone and 6-hydroxy-2-[2-(3′-methoxy-4′-hydroxyphenylethyl)] chromone (Fig. 7). The most abundant metabolites in D were 6-Methoxy-2-(2-phenylethyl) chromone and 5-Hydroxy-6-methoxy-2-(2-phenylethyl) chromone. Comparing DS with D, DS induced the accumulation of only 8-Chloro-2-(2-phenylethyl)-5,6,7-trihydroxy-5,6,7,8-tetrahydrochromone, which was consistently detected in the other three treatments (F, FS and D). Therefore, this specific compound may need further study to unravel its involvement in regulation of agarwood formation and quality attributes. Generally, the addition of brine to either F or D reduces the number of metabolites as evidenced in Table 1 and Fig. 7.

## Discussion

In spite of the fact that agarwood-producing plants such as *A. sinensis* take a long time to develop and the agarwood-formation process takes an equivalent amount of time, the demand for agarwood and its derivatives is on the rise [4, 13, 28]. To overcome this, *A. sinensis* is intentionally wounded to produce agarwood [11, 13, 37–39, 44]. The quantity and quality of agarwood is well documented to be influenced by locations and induction techniques [13, 45–47]. Thus, the present study was conducted to profile metabolome of agarwood from *A. sinensis* induced by fire and cold drill techniques with or without brine (Fig. 1). The amount of ethanol-extracted oil obtained ranged from 0.085 ± 0.035% in F to 0.143 ± 0.022% in DS (Fig. 2). Earlier studies indicated altered ethanol-extracted oil content in artificially produced agarwood from *A. sinensis* [13, 45]. The ethanol-extracted oil contents from the four treatments in the present study were higher than those reported in chemically produced agarwood [48]. Our result however compares well with similar reports by Zhang et al. [49] in *Lasiodiplodia theobromae* (Pat.). Thus, the four treatments applied in this study could offer promising techniques for improving agarwood formation. However, the present study did not assess the effect of these treatments on the quality attributes of the ethanol-extracted oil. Therefore, it is imperative that future studies assess the effects of these techniques on the agarwood quality.

Plants possess range of phenolic compounds such as flavonoids, lignins, stilbenes, tannins and lignans which are products of phenylalanine or tyrosine by-products of the shikimate pathway. From the metabolome profiling, 712 secondary metabolites comprising 122 lipids, 109 phenolic acids, 78 organic acids, 72 flavonoids, 69 amino acids and derivatives, 53 alkaloids, 41 nucleotides and derivatives, 25 terpenoids, 18 lignans and coumarins, 10 tannins and 115 unknown compounds were detected (Fig. 3A). Most of these compounds have been reported in previous studies to account for agarwood value for incense, perfume and medicine [48]. For example, catechin, epicatechin and rutin identified in this study have been reported to possess antioxidant activity and antimutagenic properties [50, 51]. These compounds were grouped into two major clusters with FS as Cluster I whereas D, DS and F formed another cluster (Fig. 3B). This trend virtually followed the same trend in the statistical analysis of ethanol-extracted oil content (Fig. 2), indicating that either of the treatments alter the metabolome profiles in the agarwood from *A. sinensis*. We further subjected the 712 compounds to differential analyses with stringent criteria. Comparing F to its counterpart with brine (FS), a total of 302 metabolites, out of which 217 were downregulated and 85 were upregulated (Fig. 4; Supplementary Table S1A). For instance, 3-Hydroxy-4′,5,7-Trimethoxyflavanone (flavonoid) as the most abundant compound in F accumulated 18.77 times higher in FS (Supplementary Table S1A). This implies that addition of brine to F largely reduced the accumulation of metabolites compared to the control in this study. Contrary, four phenolic acids ((S)-2-Hydroxy-3-(4-Hydroxyphenyl) propanoic acid; 3-(Hydroxymethyl) phenol; Dihydrocaffeic acid and 1,3-O-Di-p-Coumaroylglycerol) (VIP = 1.21–1.22; log10FC = 10.13–14.21) accumulated in FS but were completely absent in the control (F). This suggests that addition of brine has altered metabolome profile in agarwood from *A. sinensis*.

Again, we compared D_vs_DS and detected 155 differentially accumulated compounds with 94 downregulated and 61 upregulated (Fig. 4; Supplementary Table S1B). This was consistent with the trend of F_vs_FS. However, the most abundant metabolites (Nootkatone (Terpenoid)) accumulated 2.40 times lower in D than in DS (Supplementary Table S1b). Also, brine addition to cold drill induced 2-(4-Hydroxybenzyl)-4-(methoxymethyl) phenol; p-Coumaric acid-4-O-glucoside and Syringaldehyde-4-O-glucoside (phenolic acids), 2-Hydroxyisobutyric acid (organic acid), Herbacetin (flavonoid) and Methyldopa (amino acid and derivative) which were completely absent in the control (D) (Supplementary Table S1 b). This suggests that brine together with cold drill may produce agarwood with richer metabolite content.

The most prominent pathway altered among the four treatments was flavonoid/flavone and flavonol biosynthesis with 18 DAMs in pairwise comparisons (Fig. 6A). Most of the differentially enriched flavonoid compounds were in higher abundance in F than FS whereas the DS had higher abundance of differentially enriched flavonoid compounds than DS. This is not surprising as flavonoid biosynthesis has been reported in several studies on agarwood formation in *A. sinensis* [52]. Also, three metabolites (Taraxerol, Costunolide and Valencene) were differentially enriched in sesquiterpenoid and triterpenoid biosynthesis among the four treatments (Table 2; Fig. 6B). These compounds are sesquiterpenes which are reported as one of the major constituents of agarwood [53]. The abundance of these metabolites suggests that F and DS treatments may influence the quality of agarwood compared to their counterparts (FS and D, respectively). Metabolic engineering may be one of the strategies for improving agarwood quality without imposing avoidable stress on *A. sinensis* [52].

Besides, 28 metabolites consisting of alkaloids, amino acids and derivatives, flavonoids, lignans and coumarins, organic acids, phenolic acids and other compounds were explicitly involved in biosynthesis of secondary metabolites (Table 1; Fig. 6C). These can be targeted for metabolic engineering to boost agarwood production and quality. One of such compounds is salicylic acid which is reported to induce agarwood formation [54]. Basically, salicylic acid is known to induce defense response around the wounds and further to produce sesquiterpenes and phenylethyl chromones. Subsequently, this results in the formation of agarwood at the wound location [55, 56]. Cold treatment with or without brine induced six 2-(2-phenylethyl) chromones and derivatives which is contrary to the report of Naziz et al. [54]. These 2-(2-phenylethyl) chromones and derivatives have received much attention in recent years due their role in influencing quality of agarwood [57], chemical isolation [5, 58–60], pharmacological effects [61–63] and their biosynthesis pathways [19, 28]. In all, nine 2-(2-phenylethyl) chromones and derivatives were detected in F while four, six and one were detected in FS, D and DS, respectively (Fig. 7). Future study could assess the effect of the four treatments on the quality of agarwood, the type and contents of 2-(2-phenylethyl) chromones and derivatives induced. Our findings provide propelling insights to aid *A. sinensis* breeding efforts to boost agarwood production and quality.

## Conclusions

This study analyzed four methods of non-traditional agarwood production and their differential influence on oil and metabolome profile in *A. sinensis*. Our results indicate that the various treatments, fire drill, fire drill + brine treatment, cold drill and cold drill + brine treatments used in agarwood induction from *A. sinensis* had a substantial effect on the ethanol-extracted oil contents and metabolome profiles of the agarwood. Addition of brine to either fire drill or cold treatment yielded higher ethanol-extracted oil. Generally, inclusion of brine to either fire or cold drill treatments reduced the accumulation of most of the metabolites detected. The differentially accumulated metabolites detected were predominantly enriched in flavonoid/flavone, flavonol, sesquiterpenoid and triterpenoid biosynthesis. The study provides important insights into ethanol-extracted oil content and metabolome profiles of agarwood induced by four non-traditional methods which will be useful to evaluate commercial agarwood production.

## Supplementary Information

**Additional file 1: Figure S1.** Principal component analysis based on ion intensity of the metabolites.

**Additional file 2: Figure S2.** Venn diagram of differentially accumulated metabolites detected among the four pairwise group.

**Additional file 3: Table S1.** Ethanol-extracted oil content obtained from the four treatments fire drill + brine drill (FS), fire drill (F), cold drill + brine drill (DS) and cold drill (D) in quadruplicates.

**Additional file 4: Table S2.** A-D: (A) Differentially accumulated primary/secondary metabolites detected in fire drill_vs_fire + brine drill (B) Differentially accumulated primary/secondary metabolites detected in cold drill_vs_cold + brine drill, (C) Differentially accumulated primary/secondary metabolites detected in fire drill_vs_cold drill and (D) Differentially accumulated primary/secondary metabolites detected in fire + brine drill_vs_cold + brine drill in triplicates following discriminant analysis of orthogonal partial least squares with threshold of log2FC ≥ 1 and variable importance in projection (VIP) ≥ 1.

## Data Availability

Data supporting the findings of this project are in the manuscript and its supplementary files.

## Abbreviations

D: Cold Drill Treatment
DAM: Differentially Accumulated Metabolites
DS: Cold Drill + Brine Treatment
F: Fire Drill Treatment
FS: Fire Drill + Brine Treatment
GC-MS: Gas Chromatography-Mass Spectrometry
KEGG: Kyoto Encyclopedia of Genes and Genomes
OPLS-DA: Discriminant analysis of orthogonal partial least squares
PCA: Principal components analysis
PLS-DA: Partial least squares discriminant analysis
VIP: Variable importance in projection
