# Supplementary material for: Effects of various artificial agarwood-induction techniques on the metabolome of Aquilaria sinensis
Source: BMC Plant Biol. 2021 Dec 13;21:591. doi: 10.1186/s12870-021-03378-8 (PMC8667428; doi:10.1186/s12870-021-03378-8)
Supplement: Supplementary file 1 — Additional file 1: Figure S1. Principal component analysis based on ion intensity of the metabolites. [file 12870_2021_3378_MOESM1_ESM.docx]

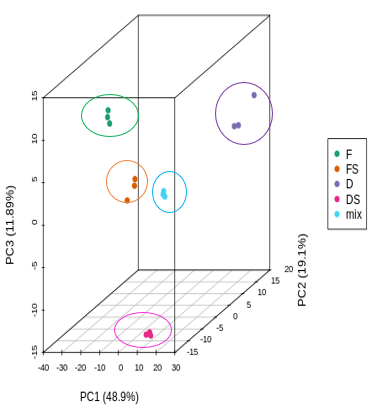


**Figure S1.** Principal component analysis based on ion intensity of the metabolites (Principal component (PC) 1, 2 and 3 accounted for 48.90, 19.10 and 11.89 % variability, respectively). Green, chocolate, medium slate blue, magenta and maroon1colors represent samples from fire drill treatment (F), fire drill + brine treatment (FS), cold drill treatment (D), cold drill + brine treatment (DS) and mix (mixture of F, FS, D and DS samples), respectively.
