# Supplementary material for: Effects of various artificial agarwood-induction techniques on the metabolome of Aquilaria sinensis
Source: BMC Plant Biol. 2021 Dec 13;21:591. doi: 10.1186/s12870-021-03378-8 (PMC8667428; doi:10.1186/s12870-021-03378-8)
Supplement: Supplementary file 2 — Additional file 2: Figure S2. Venn diagram of differentially accumulated metabolites detected among the four pairwise group. [file 12870_2021_3378_MOESM2_ESM.docx]

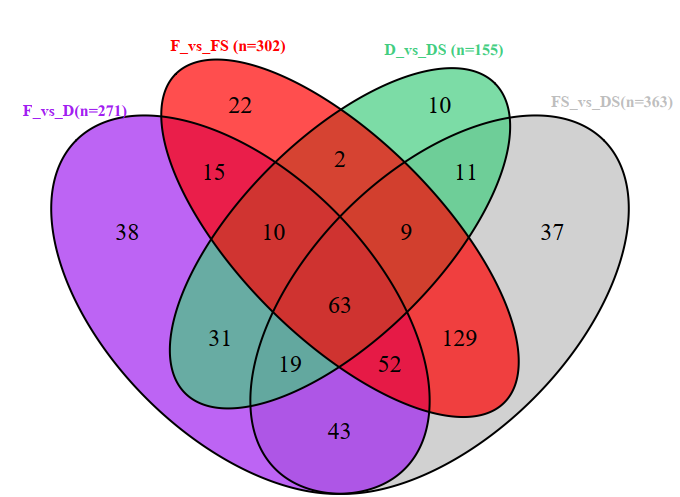


**Figure S2.** Venn diagram of differentially accumulated metabolites detected among the four pairwise group. The samples of agarwood produced from *Aquilaria sinensis* by fire drill treatment (F), fire drill + brine treatment (FS), cold drill treatment (D) and cold drill + brine treatments (DS). Number of unique metabolites detected in each treatment is represented as n.
